# The diagnostic value of magnetic resonance imaging in differentiating benign and malignant pediatric ovarian tumors

**DOI:** 10.1007/s00247-020-04871-2

**Published:** 2020-11-13

**Authors:** Carlijn L. Janssen, Annemieke S. Littooij, Marta Fiocco, Josephine C. B. Huige, Ronald R. de Krijger, Caroline C. C. Hulsker, Angelique J. Goverde, József Zsiros, Annelies M. C. Mavinkurve-Groothuis

**Affiliations:** 1 Princess Máxima Center for Pediatric Oncology, Heidelberglaan 25, 3584 CS Utrecht, The Netherlands; 2 Department of Radiology and Nuclear Medicine, University Medical Center Utrecht, Utrecht, The Netherlands; 3 Mathematical Institute Leiden University, Leiden, The Netherlands; 4 Medical Statistics, Biomedical Data Science Department, Leiden University Medical Center, Leiden, The Netherlands; 5 Department of Pathology, University Medical Center Utrecht, Utrecht, The Netherlands; 6 Department of Reproductive Medicine and Gynaecology, University Medical Center of Utrecht, Utrecht, The Netherlands

**Keywords:** Benign, Children, Magnetic resonance imaging, Malignant, Ovarian preservation, Ovaries, Tumor

## Abstract

**Background:**

The diagnostic workup of ovarian tumors in children and adolescents is challenging because preserving fertility, in addition to oncological safety, is of particular importance in this population. Therefore, a thorough preoperative assessment of ovarian tumors is required.

**Objective:**

To investigate the diagnostic value of MR imaging in differentiating benign from malignant ovarian tumors in children and adolescents.

**Materials and methods:**

We conducted a retrospective study of all children and adolescents age <18 years who underwent MR imaging of ovarian tumors during 2014–2019 at a pediatric specialty center. Two radiologists reviewed all MR imaging. We used pathology reports to define the histological diagnosis.

**Results:**

We included 30 girls who underwent MR imaging for an ovarian tumor. Signs indicative for malignancy were tumors with a diameter ≥8 cm, with areas of contrast enhancement, irregular margins, extracapsular tumor growth, and ascites. All benign and malignant ovarian tumors were correctly identified by the radiologists.

**Conclusion:**

The diagnostic utility of MR imaging in classifying ovarian tumors in children and adolescents as benign or malignant is promising and might aid in defining the indication for ovarian-sparing versus non-ovarian-sparing surgery. We recommend evaluating these tumors with MR imaging prior to deciding on surgical treatment.

## Introduction

Ovarian tumors in children and adolescents are uncommon, with an estimated incidence of 3 per 100,000 per year, of which 3.7–23.5% are malignant [1–3]. The histological types seen in childhood ovarian tumors differ significantly from the types seen in adults. In children, germ cell tumors are the most common ovarian tumors and comprise 60–90% of all pediatric ovarian malignancies, followed by sex-cord stromal (10–20%), epithelial (5–20%) and other (<5%) tumors [4, 5]. In contrast, most ovarian tumors in women are of epithelial origin [6].

Treatment should be focused on oncological safety and ovarian preservation. Patients with benign tumors can either be safely monitored or undergo a simple ovarian-sparing resection (e.g., tumorectomy or enucleation). In recent years, an increasing percentage of ovarian-sparing procedures has been reported, but many unnecessary non-ovarian-sparing surgeries (e.g., oophorectomies) are still performed in children and adolescents [7]. Therefore, a proper diagnostic evaluation of ovarian tumors is of paramount importance [4, 7, 8].

Several algorithms have been reported to identify possible ovarian malignancies before the histological diagnosis, including radiologic and tumor marker parameters. However, a single validated diagnostic method for the pediatric age group is not available [9–11]. Transabdominal ultrasonography (US) is the imaging modality of choice for initial evaluation of ovarian tumors at any age. US is useful for detecting ovarian lesions and differentiating solid, cystic and complex cystic lesions. It is the modality of choice to diagnose physiological cysts and teratomas. However, large cysts are difficult to assess completely with US and further cross-sectional imaging should be considered [12]. MR imaging is known to have several advantages compared to CT. In the pediatric population, radiation exposure by CT is of particular concern because children are inherently more radiosensitive and they have more years ahead during which radiation-induced cancer can develop [13]. Furthermore, MR imaging provides superb soft-tissue contrast resolution that increases accuracy in the diagnosis of pediatric solid tumors including ovarian lesions [14, 15]. The European Society of Urogenital Radiology (ESUR) has developed an algorithmic approach for the imaging of sonographically indeterminate ovarian tumors and recommends the use of MR [16]. This approach is based on the results of several (mainly adult) studies that have evaluated the diagnostic value of MR imaging in differentiating between benign and malignant tumors and characterizing the specific nature of ovarian tumors [17–21]. A recent systematic review showed that data evaluating the diagnostic value of MR imaging in ovarian tumors in the pediatric population are scarce [22].

The aim of this study was to investigate the diagnostic value of MR imaging in differentiating benign and malignant ovarian tumors in children and adolescents.

## Materials and methods

### Patients

All children and adolescents younger than 18 years who were diagnosed with an ovarian tumor between October 2014 and March 2019 at the Princess Máxima Center for Pediatric Oncology in Utrecht were included in this study. Patients for whom no MR images were available were excluded from the study. Demographic, clinical, radiologic, biochemical and pathological data were collected retrospectively from the medical charts. This study was approved by the local ethics committee.

### Magnetic resonance imaging

Magnetic resonance imaging at our center was performed on a 1.5-tesla (T) system (Achieva; Philips Medical Systems, Best, The Netherlands). The imaging protocol consisted of coronal and axial T2-W imaging, axial T1-W imaging with and without fat suppression, and axial T1-W imaging after gadolinium contrast medium administration. Diffusion-weighted imaging (DWI) was acquired in axial plane during free breathing, with b values of at least 0 s/mm^2^, 100 s/mm^2^ and 1,000 s/mm^2^ (Table 1). Depending on the ability to cooperate, children were awake or under general anesthesia. No oral contrast agents were used. All children were screened for MR contraindications.

**Table 1:** Scan parameters at 1.5-T MRI for abdominal tumors

Parameter	T2-weighted TSE	3-D T2-weighted TSE	Diffusion-weighted imaging	T1-weighted imaging pre/post gadolinium injection
Pulse sequence	2-D turbo spin echo	3-D turbo spin-echo with variable flip angle	2-D single-shot spin echo with spectral fat saturation	2-D ultrafast spoiled gradient echo with fat-suppression
Repetition time (ms)	6,667	447	1,946	5.50
Echo time (ms)	100	90	76	2.70
Slice orientation	Axial	Coronal	Axial	Axial
Slice thickness (mm)	4	1.15	5	3
Slice gap (mm)	0	0	0	1
Echo train length	25	85	35	60
Acquisition matrix	332×262	348×348	88×70	232×233
b values (s/mm^2^)	–	–	At least 0, 100, 1,000	–

*TSE* turbo spin echo

Fifteen girls (Table 2) underwent MR imaging at another hospital. The MR imaging protocol varied among these girls, but all 15 had at least a T1-W and T2-W sequence performed, with a maximum slice thickness of 5 mm for T1-W images and 4 mm for T2-W images.

**Table 2:** Clinical characteristics of the included patients based on histological type

		Total	Histological type		
			Benign	Borderline	Malignant
Number of cases		30	15 (50%)	2 (6.7%)	13 (43.3%)
Age of patient (y) (mean ± SD)		11.9±4.0	10.5±4.5	15.6±1.7	12.9±3.0
Menarcheal status	Premenarcheal	16	9	0	7
	Postmenarcheal	13	5	2	6
	Unknown	1	1	0	0
Presentation of symptoms	Symptomatic	27	12	2	13
	Asymptomatic	3	3	0	0
Duration of symptoms	Acute	19	9	1	9
	Chronic	11	6	1	4
Tumor markers^a^	Normal	15	14	1	0
	Abnormal	15	1	1	13
Alpha-fetoprotein^a^	Normal	20	14	2	4
	Abnormal	9	0	0	9
	Unknown	1	1	0	0
Beta-human chorionic gonadotropin^a^	Normal	27	14	2	11
	Abnormal	2	0	0	2
	Unknown	1	1	0	0
Lactate dehydrogenase^a^	Normal	17	11	1	5
	Abnormal	8	0	0	8
	Unknown	5	4	1	0
Cancer antigen 125^a^	Normal	13	10	1	2
	Abnormal	13	1	1	11
	Unknown	4	4	0	0
Inhibin-B^a^	Normal	14	9	2	3
	Abnormal	2	0	0	2
	Unknown	14	6	0	8

*SD* standard deviation, *y* years

^a^According to the age-dependent cut-off values of our center

### Radiologic assessment

The MR images were retrospectively evaluated by two independent reviewers (A.S.L. and J.C.B.H., with 10 and 2 years of experience in pediatric abdominal MR imaging, respectively). They were unaware of the girls’ clinical characteristics and pathological results. The two radiologists scored all images independently according to a set of radiologic items (Table 3). They measured the size of the tumors and classified the morphological appearance (predominantly cystic, cystic and solid, or predominantly solid). They evaluated signal intensity features (e.g., T1 and T2 signal intensity) and contrast enhancement. Tumor margins, extracapsular growth, ascites and locoregional and distant tumor spread (enlarged lymph nodes, peritoneal deposits, distant metastasis) were scored individually. Ascites was defined as free fluid in and beyond the paracolic gutter. Apparent diffusion coefficient (ADC) values (10^−3^ mm^2^/s) of the pathological lesions were measured three times in a circular region of interest in the enhancing parts of the lesions. Finally, tumors were assessed as “radiologically benign” or “radiologically malignant” by the two radiologists in consensus. The following items were considered indicative of malignancy: irregular margins, extra-capsular growth, peritoneal deposits, enlarged lymph nodes and distant metastasis, all tumors with no fat or calcified component (indicative for germ cell origin) and enhancing components, and all tumors with large enhancing parts. Signs indicative of benign lesions were tumors with no solid enhancing components, tumors with typical imaging features of teratoma with only small proportion of enhancing components, and tumors with very low T2 signal (indicative for fibrothecoma).

**Table 3:** Diagnostic performance of MRI in differentiating benign and malignant pediatric ovarian tumors

Parameters		Histological type		Sensitivity (95% CI)	Specificity (95% CI)
		Benign (*n*=15)	Malignant (*n*=13)		
Radiologic assessment	Radiologically benign	15	0	100 (78,100)	100 (75,100)
	Radiologically malignant	0	13		
Largest diameter of tumor (cm)	≤8 cm	8	0	53 (27,79)	100 (75,100)
	≥8 cm	7	13		
Morphological appearance	Predominantly cystic	10	0	66 (38,88)	100 (75,100)
	Partly or predominantly solid	5	13		
High T1 signal	Absent or missing	2	1	17 (2,48)	89 (52,100)
	Present	10	8		
	Missing	3	4		
Signs for fat consisting high T1 signal	Yes	12	3	80 (52,96)	75 (43,95)
	No	3	9		
	Missing	0	1		
T2 signal intensity of solid components	Hypointensity and/or isointensity and/or no solid components present	8	0	53 (27,79)	100 (75,100)
	Hyperintensity and/or variable over different areas	7	13		
Contrast enhancement of solid components	Absent	10	0	67 (38,88)	100 (75,100)
	Present	5	13		
Diameter of enhancing components (cm)	Enhancement absent or ≤8 cm	13	1	87 (60,98)	92 (64,100)
	≥8 cm	2	12		
Margin	Regular	15	2	100 (78,100)	85 (55,98)
	Irregular	0	11		
Extracapsular tumor growth	Absent	15	6	100 (78,100)	54 (25,81)
	Present	0	7		
Enlarged lymph nodes	Absent	15	12	100 (78,100)	8 (0,36)
	Present	0	1		
Ascites	Absent	13	0	87 (60,98)	100 (75,100)
	Present	2	13		
Peritoneal deposits	Absent	15	10	100 (78,100)	25 (5,54)
	Present	0	3		
Distant metastasis	Absent	15	13	100 (78,100)	0 (0,25)
	Present	0	0		

*CI* confidence interval

### Pathological analysis

We used original pathology reports to define the histological diagnosis in this study. All histological diagnoses were established or reviewed (after referral) at our center. All cases were classified according to the World Health Organization classification [23].

For this analysis the different histological types were clustered into three groups: benign, borderline and malignant. Mature teratomas, fibrothecomas and grade 1 immature teratomas (but not higher grade) were clustered with the benign group. Borderline epithelial ovarian tumors were clustered in a separate group. Immature teratoma grades 2 and 3, Sertoli–Leydig cell and granulosa cell tumors were clustered with the malignant group because these tumors demonstrate a spectrum of potentially malignant behavior.

### Statistical analysis

Analysis was performed to assess the accuracy of MR imaging for determining the nature of the ovarian tumors compared to the definitive histological diagnosis. Descriptive statistics were provided for continuous variables as mean and standard deviation and for categorical variables the number in each category. Kappa statistics (defined as: poor [κ 0–0.2], fair [κ 0.21–0.40], moderate [κ 0.41–0.60], good [κ 0.61–0.80] and very good [κ >0.81]) were used to assess the interobserver variability. To assess the performance of radiologic assessment, we computed sensitivity and specificity, as well as the exact binomial 95% confidence interval for sensitivity and specificity [24]. SPSS version 25 (IBM, Armonk, NY) was employed for the statistical analysis. Statistical significance was set at *P*<0.05.

## Results

### Study population and clinical characteristics

**Fig. 1:**
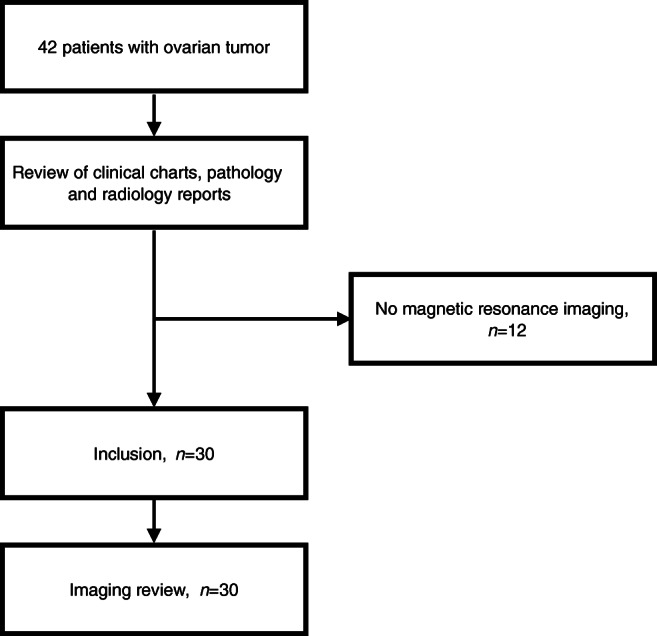
Flow chart shows the inclusion of patients

Thirty children were included into our study (Fig. 1). Clinical characteristics and MR imaging were reviewed. Clinical characteristics of the included patients are shown in Table 2. The ovarian tumors comprised 15 benign tumors, 2 borderline tumors and 13 malignant tumors. The mean age of the two girls with borderline ovarian tumors is slightly higher than that of the other children. All malignant ovarian tumors showed abnormal tumor markers.

### Radiologic assessment

The final radiologic assessment into benign versus malignant was based on consensus between the radiologists. In the assessment of different items, kappa values of interobserver agreement ranged between 0.283 and 0.839. The presence of high T1 signal was a difficult criterion to review (kappa 0.369) because of the frequent absence of a T1-sequence without fat suppression (*n*=7). In three cases, no contrast agent was used, which made assessment of enhancement impossible.

**Fig. 2:**
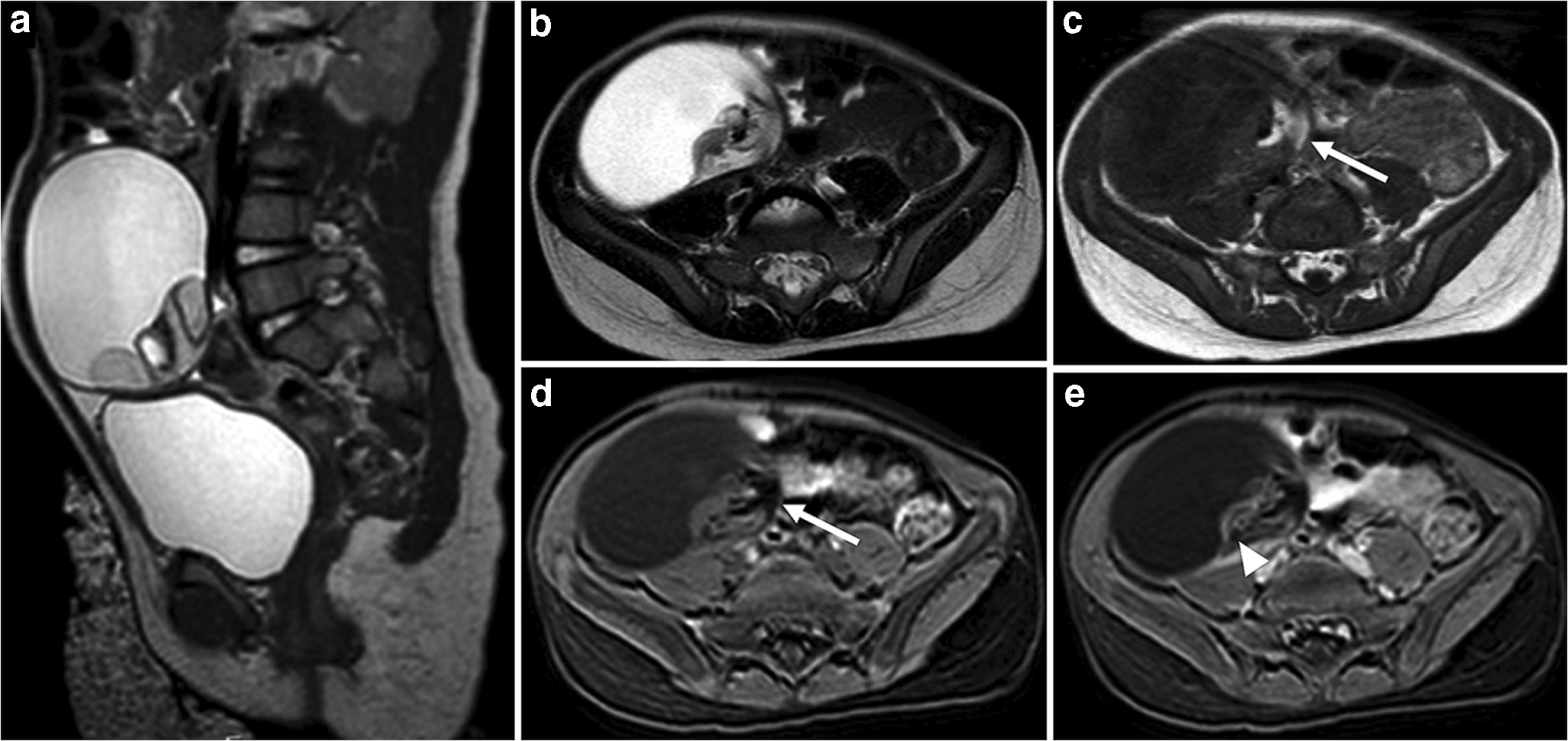
Mature teratoma in a 2-year-old girl with palpable abdominal mass. **a, b** Sagittal (**a**) and axial (**b**) T2-weighted MR images show right ovarian mass, predominantly cystic with a solid component in its wall. **c, d** Axial T1-weighted image (**c**) shows a hyperintense component (*arrows*) that demonstrates signal loss at the T1-weighted images, with fat-saturation (**d**) consistent with a fatty component. **e** After gadolinium contrast administration, there is only a small component of enhancement (*arrowhead*). These imaging features are suggestive of a mature teratoma, which was confirmed by histopathology

**Fig. 3:**
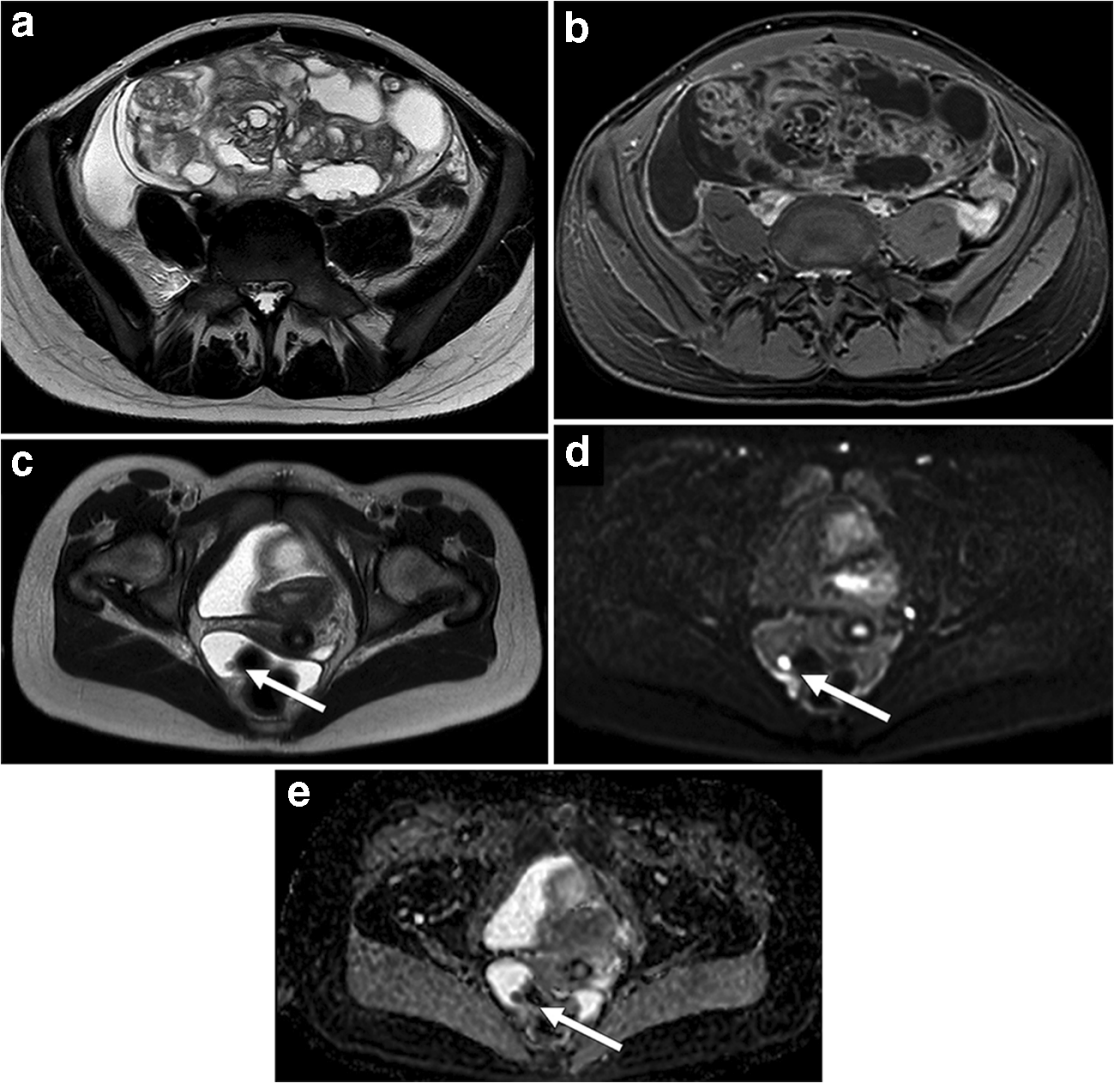
Immature teratoma in a 15-year-old girl with increasing abdominal girth. **a** Axial T2-weighted MR image shows a tumor arising from left ovary with ascites. The tumor consists of solid and cystic components. **b** Axial T1-weighted fat-saturated post-contrast MR image shows large components with enhancement. **c** Axial T2-weighted MR image in the lower pelvis demonstrates a peritoneal nodule (*arrows*). **d**, **e** The nodule has high signal at b 1,000 diffusion-weighted imaging (**d**) and low values on the corresponding apparent diffusion coefficient map (**e**), suspicious for a peritoneal deposit. The ascites combined with the peritoneal deposit are suggestive for a malignant ovarian tumor. The histopathology result after resection showed an immature teratoma, grade II, with peritoneal deposits

Imaging examples are shown in Figs. 2 and 3. Several individual MR imaging criteria were indicative for malignancy (Table 3). A threshold of ≥8 cm in tumor size for malignancy has a positive predictive value of 65% and a negative predictive value of 100%. Contrast enhancement can be seen in benign (*n*=5) and malignant (*n*=13) ovarian tumors. When applying the same threshold of ≥8 cm in diameter of enhancing solid components on MR imaging as a risk factor for malignancy preoperatively, the positive and negative predictive values are 85.7% and 92.9%, respectively. Furthermore, indicative for malignancy were the presence of an irregular margin, extracapsular tumor growth and ascites.

The diagnostic performances of the final radiologic assessment of benign or malignant, as well as the separate imaging features, are shown in Table 3. The radiologists identified 13 of 13 histologically malignant ovarian tumors as radiologically malignant and 15 of 15 benign tumors as radiologically benign. Examples of a benign and a malignant ovarian tumor are shown in Figs. 2 and 3.

We were able to measure ADC values in 14 girls. Three girls with a benign ovarian tumor had ADC values ranging 0.974–1.255×10^−3^ mm^2^/s, six girls with a malignant ovarian tumor had ADC values ranging 0.729–1.509×10^−3^ mm^2^/s and one girl with a borderline tumor had an ADC value of 1.683×10^−3^ mm^2^/s. There was a considerable overlap in ADC values between benign and malignant tumors. In our limited cohort, we were not able to find a correlation between ADC values and malignancy.

The two borderline tumors showed very different radiologic behavior. One borderline tumor had a predominantly cystic morphological appearance and a large diameter of 27.2 cm but showed no other abnormalities. The radiologists classified this tumor as radiologically benign. The other borderline tumor showed a diameter of 15.6 cm and predominantly cystic morphological appearance with papillary projections in the tumor wall. There were contrast enhancement of these papillary projections, ascites and peritoneal deposits. This tumor was classified by the radiologists as radiologically malignant. The pathological diagnoses of the borderline tumors were serous and mucinous borderline tumor, respectively.

## Discussion

This study shows that MR imaging can be of additional value to differentiate benign from malignant ovarian tumors in children and adolescents. Signs indicative for malignancy are tumors with a diameter ≥8 cm, with areas of contrast enhancement, irregular margins, extracapsular tumor growth and ascites.

In adults, MR imaging of sonographically indeterminate ovarian tumors is included in the ESUR guidelines for ovarian tumors [16]. The diagnostic performance of MR imaging in adults has a fairly good sensitivity for differentiating malignant from benign ovarian tumors, but regarding specificity there is room for improvement (sensitivity and specificity varied 84.8–100% and 20.0–98.4%, respectively) [22]. In adult studies the MR imaging criteria advocated as best predictive of malignancy are larger size, solid components demonstrating contrast enhancement, ascites and peritoneal deposits [25–28].

The ESUR guidelines recommend contrast-enhanced T1-W imaging for assessing ovarian tumors in adults with a morphological appearance suspicious for malignancy, e.g., with solid components within cystic tumors or nodular or irregular thickening of the outer aspects of the wall of a mass [16]. However, our study showed a weak distinctiveness of contrast enhancement for differentiating benign and malignant ovarian tumors because 33% of our benign tumors also showed contrast enhancement. This difference in distinctiveness of contrast enhancement might be explained by the significant different histological distribution of ovarian tumors in children. In contrast to adults, germ cell tumors, benign and malignant combined, are the most common ovarian tumors in children and often show contrast enhancement [28]. This complicates the radiologic differentiation between benign and malignant tumors in children. Our study showed that a larger diameter (≥8 cm) of enhancing components can help, but further studies including larger cohorts are needed to validate our findings.

In our study, there was a considerable overlap between the mean ADC values of the solid components for the benign lesions when compared to those of the borderline and malignant lesions. This lack of significance might be attributed to the rather small number of children with available ADC measurements because of the retrospective nature of our study. Benign tumors with a dense composition that is not the consequence of increased cellularity but rather of the presence of keratinoid substances, products of hemoglobin degradation and dense fibers, including mature teratomas and fibromas, are at risk of false-positive findings [22]. Further studies including a larger number of selected pathological conditions focusing on enhancing components of solid or complex tumors are warranted to explore where DWI might be advantageous with regard to ovarian tumor characterization.

Several studies have shown that MR imaging can improve the accuracy of the diagnostic workup of ovarian tumors and might even guide surgical strategy [18–21]. In the treatment of ovarian tumors, especially in children, adolescents and young adults, oncological principles — safety on one side and preservation of fertility on the other — have to be very well balanced. An accurate diagnostic workup of ovarian tumors that can identify possible ovarian malignancies before the histological diagnosis is useful. Besides radiologic characteristics, tumor markers are important in the diagnostic workup to differentiate malignant from benign ovarian tumors.

MR imaging as part of the diagnostic workup of ovarian tumors can aid in determining the surgical strategy [18–21]. Given that surgery is the primary treatment for ovarian tumors, ovarian salvage with fertility preservation is an important surgical consideration when managing children and adolescents with ovarian tumors. Benign ovarian tumors should be treated with ovarian-sparing techniques. However, several factors can ultimately influence the decision of the surgeon. Whether the intended ovarian-sparing surgical strategy is feasible can only be established during the actual surgery. The main reasons for failure of ovarian-sparing treatment are lack of residual healthy ovarian tissue, difficulty in identifying the anatomical plane between tumor and healthy tissue, and lack of healthy ovarian tissue between the tumor and fallopian tube.

In girls with a malignant ovarian mass, the primary goal is cure. However, the potential for fertility as well as future hormonal health must also be considered. The American Pediatric Surgical Association (APSA) has provided recommendations based on literature review for fertility preservation in children with malignant ovarian tumors. The APSA found that cystectomy alone in the setting of immature or malignant germ cell tumors is not supported by the current literature, and is not considered standard of care, even when platinum-based chemotherapy is given. In girls with borderline ovarian tumors, however, cystectomy might be considered because recurrence can be salvaged with additional surgery with a limited effect on survival [14]. There is a need for prospective studies to investigate the safety of ovarian-sparing surgery in malignant ovarian tumors. It would also be interesting to study, in a large cohort, whether borderline tumors can be accurately identified by MR imaging.

There are some limitations to this study. We are a pediatric oncological center and patients are only referred to our institution with a suspected malignancy. As a consequence, complex and suspicious tumors are more likely to be seen at our center. That might explain why our cohort contains more malignant ovarian tumors (43.3%) than expected in a normal population (3.7–23.5%) [2, 3]. The retrospective design inevitably causes inclusion bias and missing data. Also, the small sample size is a limitation of our study. The heterogeneity of the MR imaging protocol in our population made it more difficult to assess all items on the radiologic assessment. However, the results are promising to continue with further prospective research.

## Conclusion

The diagnostic ability of MR imaging in classifying ovarian tumors in children and adolescents as benign or malignant is promising and might help in defining the indication for ovarian-sparing versus non-ovarian-sparing surgery. Therefore, the addition of MR imaging in the diagnostic workup of pediatric ovarian tumors is recommended.

## References

[1] Skinner MA, Schlatter MG, Heifetz SA, Grosfeld JL (1993). Ovarian neoplasms in children. Arch Surg.

[2] Spinelli C, Pucci V, Strambi S (2015). Treatment of ovarian lesions in children and adolescents: a retrospective study of 130 cases. Pediatr Hematol Oncol.

[3] Freud E, Golinsky D, Steinberg RM (1999). Ovarian tumors in children. Clin Pediatr.

[4] Hermans AJ, Kluivers KB, Janssen LM (2016). Adnexal tumors in children, adolescents and women of reproductive age in the Netherlands: a nationwide population-based cohort study. Gynecol Oncol.

[5] Brookfield KF, Cheung MC, Koniaris LG (2009). A population-based analysis of 1,037 malignant ovarian tumors in the pediatric population. J Surg Res.

[6] Quirk JT, Natarajan N, Mettlin CJ (2005). Age-specific ovarian cancer incidence rate patterns in the United States. Gynecol Oncol.

[7] Papic JC, Finnell SME, Slaven JE (2014). Predictors of ovarian malignancy in children: overcoming clinical barriers of ovarian preservation. J Pediatr Surg.

[8] Hermans AJ, Kluivers KB, Wijnen MHWA (2015). Diagnosis and treatment of adnexal tumors in children and adolescents. Obstet Gynecol.

[9] Lawrence AE, Gonzalez DO, Fallat ME (2019). Factors associated with management of pediatric ovarian neoplasms. Pediatrics.

[10] Depoers C, Martin FA, Timoh KN (2019). A preoperative scoring system for adnexal mass in children and adolescents to preserve their future fertility. J Pediatr Adolesc Gynecol.

[11] Renaud EJ, Sømme S, Islam S (2019). Ovarian masses in the child and adolescent: an American pediatric surgical association outcomes and evidence-based practice committee systematic review. J Pediatr Surg.

[12] Levine D, Brown DL, Andreotti RF (2010). Management of asymptomatic ovarian and other adnexal cysts imaged at US Society of Radiologists in Ultrasound consensus conference statement. Ultrasound Q.

[13] Pearce MS, Salotti JA, Little MP (2012). Radiation exposure from CT scans in childhood and subsequent risk of leukaemia and brain tumours: a retrospective cohort study. Lancet.

[14] Jeong YY, Outwater EK, Kang HK (2000). Imaging evaluation of ovarian masses. Radiographics.

[15] Voss SD (2018). Staging and following common pediatric malignancies: MRI versus CT versus funcional imaging. Pediatr Radiol.

[16] Forstner R, Thomassin-Naggara I, Cunha TM (2017). ESUR recommendations for MR imaging of the sonographically indeterminate adnexal mass: an update. Eur Radiol.

[17] Zhang H, Zhang GF, He ZY (2014). Prospective evaluation of 3T MRI findings for primary adnexal lesions and comparison with the final histological diagnosis. Arch Gynecol Obstet.

[18] Mohaghegh P, Rockall AG (2012). Imaging strategy for early ovarian cancer: characterization of adnexal tumors with conventional and advanced imaging techniques. Radiographics.

[19] Masch WR, Daye D, Lee SI (2017). MR imaging for incidental adnexal mass characterization. Magn Reson Imaging Clin N Am.

[20] Foti PV, Attinà G, Spadola S (2016). MR imaging of ovarian masses: classification and differential diagnosis. Insights Imaging.

[21] Vargas HA, Barrett T, Sala E (2013). MRI of ovarian masses. J Magn Reson Imaging.

[22] Nimwegen van LWE, Mavinkurve-Groothuis AMC, Krijger de RR et al (2019) MR imaging in discriminating between benign and malignant pediatric ovarian masses: a systematic review. Eur Radiol 30:1166–1181 10.1007/s00330-019-06420-4 PMC6957553 31529256

[23] Meinhold-Heerlein I, Fotopoulou C, Harter P (2016). The new WHO classification of ovarian, fallopian tube, and primary peritoneal cancer and its clinical implications. Arch Gynecol Obstet.

[24] Deeks JJ, Altman DG (1999). Sensitivity and specificity and their confidence intervals cannot exceed 100%. BMJ.

[25] Hricak H, Chen M, Coakley FV (2000). Complex adnexal masses: detection and characterization with MR imaging — multivariate analysis. Radiology.

[26] Valentini AL, Gui B, Micco M (2012). Benign and suspicious ovarian masses — MR imaging criteria for characterization: pictorial review. J Oncol.

[27] Jun SE, Lee JM, Rha SE (2002). CT and MR imaging of ovarian tumors with emphasis on differential diagnosis. Radiographics.

[28] Cecchetto G (2014). Gonadal germ cell tumors in children and adolescents. J Indian Assoc Pediatr Surg.

